# Challenges in inbreeding estimation of large populations based on Polish Holstein-Friesian cattle pedigree

**DOI:** 10.1007/s13353-018-0441-x

**Published:** 2018-04-11

**Authors:** Ewa Sell-Kubiak, Łukasz Czarniecki, Tomasz Strabel

**Affiliations:** 1 Centre for Genetics, Polish Federation of Cattle Breeders and Dairy Farmers, Poznań, Poland; 2 Department of Genetics and Animal Breeding, Poznan University of Life Sciences, Poznan, Poland

**Keywords:** Dairy cattle, Pedigree completeness, Population structure, Relatedness

## Abstract

The aim of this study was to evaluate observed and future inbreeding level in Polish Holstein-Friesian cattle population. In total, over 9.8 mln animals were used in the analysis coming from the pedigree of Polish Federation of Cattle Breeders and Dairy Farmers. Inbreeding level, as an average per birth year, was estimated with the method accounting for missing parent information with the assumption of year 1950 as the base year of the population. If an animal had no ancestral records, an average inbreeding level from its birth year was assigned. Twice the average inbreeding level served as relatedness of the animal to the population, which enabled estimation of inbreeding in its offspring. The future inbreeding of potential offspring was estimated as an average of animals (bulls and cows) available for mating in a certain year. It was observed that 30–50% of animals born between 1985 and 2015 had no relevant ancestral information, which is caused by a high number of new animals and/or entire farms entering the national milk recordings. For the year 2015, the observed inbreeding level was 3.30%, which was more than twice the inbreeding with the classical approach (without missing parent information) and higher by 0.4% than the future inbreeding. The average increase of inbreeding in years 2010–2015 was 0.10%, which is similar to other countries monitored by World Holstein-Friesian Federation. However, the values might be underestimated due to low pedigree completeness. The estimates of future inbreeding suggested that observed inbreeding could be even lower and also increase slower, which indicates a constant need to monitor rate of increase in inbreeding over time. The most important aspect of presented results is the necessity to advise individual farmers to keep precise recordings of the matings on their farm in order to improve the pedigree completeness of Polish Holstein-Friesian and to use suitable mating programs to avoid too rapid growth of inbreeding.

## Introduction

Despite the worldwide character of Holstein-Friesian cattle population, its inbreeding level is, reportedly, continuously increasing. It is partially caused by progressing globalization of dairy cattle breeding, which leads to using a similar gene pull across all the populations. Thus, the control of inbreeding level in Holstein-Friesian cattle populations is currently one of the main focuses of World Holstein-Friesian Federation (WHFF; Feddersen et al. 2016). Increase in inbreeding level leads to occurrence of inbreeding depression (Rokouei et al. 2010; Lal et al. 2013) and a decrease in genetic variation among the animals (VanRaden 2005). The presence of inbreeding depression is associated with decrease in vitality and higher susceptibility to diseases and unfavorable environmental conditions. As a consequence, a lower reproductive (e.g., low insemination success, miscarriages) and production performance as well as higher rate of genetic disorders and lethal genes are observed. Whereas the decrease in genetic variation leads to slowing down or even completely stopping of the genetic progress of traits under selection, which is a consequence of increasing homozygosity level leading also to higher rate of a lethal diseases. Increase of inbreeding level in the population of a country or mating highly related animals within one herd will cause actual economic losses. Therefore, the WHFF promotes the control of rate of increase in inbreeding over time in worldwide populations of Holstein-Friesian cattle (Feddersen et al. 2016) and not exceeding the increase of 1% (Falconer and Mackay 1996; Lynch and Walsh 1998) per generation (~4 years).

Poland is one of the countries where monitoring of inbreeding is currently being introduced. Currently, in Poland, around 36% of Holstein-Friesian cows are under genetic evaluation, but this number increases each year with new animals entering the national milk recordings (“The results of milk recordings” 2016; http://pfhb.pl). The pedigree information of the newly added cows is scarce. Therefore, to be able to estimate the actual inbreeding level in this population, it is necessary to use a method allowing to account for information from the “missing ancestors” as the traditional approach of Wright (1922) is no longer enough. Such methodology was developed by VanRaden (1992), who proposed to assign average inbreeding level from the year of birth of individuals if their pedigree had no ancestors or only one parental line available. This “assigned inbreeding level” is later used to estimate the inbreeding of the offspring. Since the inbreeding level of the animal is equal half of its additive relationship to the population, it allows estimation of the relatedness of an animal to the population. Another important aspect that can serve as a tool in monitoring the inbreeding level in the population is estimation of a future inbreeding (VanRaden and Smith 1999). The future inbreeding represents the expected level of inbreeding in the population, based on inbreeding of animals currently available for matings. It indicates the difference between the observed inbreeding level and the inbreeding that would be present without preferences for a certain animals.

Thus, the objective of this study was to estimate the observed and future inbreeding level in Polish Holstein-Friesian cattle population.

## Materials and methods

### Data evaluation

The pedigree data for this study came from the Polish Federation of Cattle Breeders and Dairy Farmers (Warsaw, Poland) collected in the course of national milk recordings of Polish Holstein-Friesian cattle population. The original Polish pedigree dataset covered information of 10,072,784 animals born from year 1899 to 2016. During the data evaluation, a total of 271,935 animals were removed (see Table 1 for more details). The pedigree was also tested for a list of other aspects; however, those did not lead to removing records from the dataset.

**Table 1:** Data control of the pedigree of bulls and cows with the number of animals kept for inbreeding evaluation in Polish Holstein-Friesian population

Pedigree control	Number of excluded animals	Final number of animals
Removing a parent if offspring younger by less than 3 years	17,006	9,800,849
Removing dams with more than 14 calves	1808	
Removing animals that are present both as dam and sire and sex cannot be verified	3	
Removing animals with incorrect IDs (less than six digits without country code or less than seven digits with country code)	253,124	

All animals from the corrected pedigree had also information on the year of birth, which was needed for the further calculations of inbreeding level per year. If the data provided by the Polish Federation of Cattle Breeders of Dairy Farmers did not contained the year of birth of the animal, it was calculated using the assumption that the animal had to be 3 years older than the youngest offspring present in the dataset (VanRaden 1992; Aguilar and Misztal 2008).

### Establishing the base year

The study of VanRaden (1992) suggested to use the base year when calculating the inbreeding level with accounting for missing parent information as the values of calculated inbreeding coefficients depended on the pedigree depth. This was done in order to have better control of the animals included in the evaluation and to avoid overestimation of inbreeding level per birth year, when only a few animals had the pedigree available in early years of the start of population. In case of Polish population, it was decided to use animals born not earlier than in 1950 as from this year the number of Holstein-Friesian in Poland cattle began to increase intensively and historically the status of the population became more stable after Second World War (Jasiorowski et al. 1988).

After defining the base year, certain steps had to be taken to edit the pedigree for the analysis (VanRaden 1992):

1. Animals born before 1950 were kept in dataset if they had at least two offspring born after 1950;
2. Parents born before 1950 were treated as unrelated and not inbred;
3. Animals born as a first generation after 1950 were treated as related but not inbred;
4. Animals born in a second and later generations had increasing level of relatedness and inbreeding.

### Calculating pedigree completeness

Pedigree completeness for each individual in the pedigree was estimated with the use of pedigree completeness index based on five generations (PCI-5; MacCluer et al. 1983) following the equation:

$$ \mathrm{PCI}=\frac{2{C}_f\ast {C}_m}{2{C}_f+{C}_m}, $$

where *C*_*f*_ and *C*_*m*_ are the proportions of paternal and maternal ancestors in the pedigree estimated based on the equation:

$$ C=\frac{1}{d}{\sum}_{i=1}^d{a}_i, $$

where *a*_*i*_ is the ratio of known to unknown ancestors in each generation and *d* is the number of generation. The PIC-5 is one of the most commonly used methods to evaluate pedigree completeness, which enables comparison with other studies and populations worldwide (Kearney et al. 2004; Sørensen et al. 2005; Stachowicz et al. 2011).

The value of PCI-5 for each animal could have the value from 0 to 1, depending on the number of “full generations,” where maximum value of 1 means that the animal had five full generations recorded in the pedigree. It should be noted that the first value larger than zero was PCI-5 = 0.125, which meant that the animal had at least two parents and two grandparents (each from different parent). The value of PCI-5 = 0 was, thus, assigned to all animals with less than 1.5 of full generation in pedigree, and accounted for more cases than purely “animals without parents.”

### Estimation of observed inbreeding

The observed inbreeding level per year was estimated based on method of Emik and Terrill (1949) with extension of accounting for missing parents proposed by VanRaden (1992) and using the algorithm adjusted by Aguilar and Misztal (2008). The method consisted of a few steps. Firstly, the average inbreeding level per birth year was estimated by using the tabular method of Emik and Terrill (1949):

$$ {\mathrm{F}}_{\mathrm{x}}=0.5{\mathrm{R}}_{\mathrm{sd}}, $$

where *R*_sd_ is the relationship between sire and dam of the animal.

Calculation of each *R*_sd_ requires screening the pedigree in search of ancestors of sire and dam to estimate the relationship between them considering three cases (Aguilar and Misztal 2008):

$$ {\mathrm{R}}_{\mathrm{x}\mathrm{y}}=\left\{\begin{array}{cc}0& \mathrm{if}\kern0.5em \mathrm{x}=0\kern0.5em \mathrm{or}\kern0.5em \mathrm{y}=0\\ {}1+{\mathrm{F}}_{\mathrm{x}}& \mathrm{if}\kern0.5em \mathrm{x}=\mathrm{y}\\ {}0.5\left({\mathrm{R}}_{\mathrm{sy}}+{\mathrm{R}}_{\mathrm{dy}}\right)& \mathrm{otherwise}\end{array},\right. $$

where animal *x* has to be younger than animal *y* and sire and dam are parents of animal *x*.

The first condition with *x* or *y* equal to 0 is an indication of unknown parent/parents of the animal *x*. Extension of this algorithm to include a non-zero inbreeding coefficient of unknown parents is done by giving a negative value of birth year of animal *x* or *y* to offspring of those parents (Aguilar and Misztal 2008). Thus, the first condition being *R*_*xy*_ = 0 is transformed to

$$ {\mathrm{R}}_{\mathrm{xy}}=2\left\{\begin{array}{cc}{\mathrm{b}}_{\hbox{-} \mathrm{x}}& \mathrm{if}\kern0.5em \mathrm{x}<0\kern0.5em \mathrm{and}\kern0.5em \mathrm{y}>0\\ {}{\mathrm{b}}_{\hbox{-} \mathrm{y}}& \mathrm{if}\kern0.5em \mathrm{x}<0\kern0.5em \mathrm{and}\kern0.5em \mathrm{y}>0\\ {}\max \left({\mathrm{b}}_{\hbox{-} \mathrm{x}},{\mathrm{b}}_{\hbox{-} \mathrm{y}}\right)& \mathrm{if}\kern0.5em \mathrm{x}<0\kern0.5em \mathrm{and}\kern0.5em \mathrm{y}>0\end{array}\right., $$

where *b* is the average inbreeding coefficient for all animals born in a certain year (Aguilar and Misztal 2008). The calculation of *b* is done iteratively, so that its values are estimated each round with *b* = 0 in the first iteration (Aguilar and Misztal 2008). Only animals with at least both parents were used for this estimation (VanRaden 1992).

Secondly, the obtained average inbreeding was assigned to all animals from a certain birth year that did not have parents or only mother or father were present in the dataset. Finaly, the assigned values were used as a rate of relatedness of the individual to the population. It was assumed that twice the inbreeding of the animals with missing pedigree was expressing its relationship to the population. This was later used for more accurate estimations of inbreeding level in offspring (VanRaden 1992).

### Estimation of future inbreeding

The future inbreeding was estimated to verify whether the level of the observed inbreeding and its increase rate follow the average relatedness in the population. The selection of the animals was based on work of VanRaden and Smith (1999) and adjusted for the Polish population. The level of the future inbreeding for each year was estimated as an average value of inbreeding of animals that could potentially be available in the population for matings. The calculation of the future inbreeding for year 2016 based on data from 2010 to 2015 was as follows:

1. Selecting the bulls:
  - Not older than 5 years (birth year 2011–2015);
  - Older than 5 years if the last use was more recent than 5 years.
2. Selecting the cows:
  - Cows not older than 6 years (birth year 2010–2014);
  - Pooling 200 cows per birth year—1000 cows in total for a 5-year period.
3. “Mating” of selected animals and estimating the average inbreeding for potential offspring per birth year.

## Results and discussion

The inbreeding level in any population of livestock animals is a direct consequence of selection. It is an ultimate challenge of breeding strategies to maximize response to selection without facing the consequences of increasing inbreeding level in the population (Strabel 2001). However, the estimated inbreeding level is affected by many factors not only directly linked to selection and breeding practices (e.g., selection intensity, semen, and animal import), but also pedigree completeness level, establishing or not a base year for the population or method used for its estimation. Thus it is necessary to constantly monitor and thoroughly examine the results as the increasing level of inbreeding is causing economic losses (Strabel 2001). Following the recommendations of WHFF and comprehensive studies performed in other local populations of Holstein-Friesians, e.g., UK (Kearney et al. 2004), Denmark (Sørensen et al. 2005), Canada (Miglior and Burnside 1995), and USA (Young and Seykora 1996), the Polish population also required similar evaluation. This study was conducted to evaluate the pedigree and inbreeding level in Polish Holstein-Friesian cattle population, which was performed with a method enabling to account for missing parent information.

### Pedigree data evaluation

The foundation of Polish Holstein-Friesian population can be dated back to early 1900s, yet, only from 1934, with implementation of studbook regulations, all information on milk production and pedigree started to be recorded in a uniform manner (Jasiorowski et al. 1988; Goździkiewicz, 2004). Due to losing nearly 67% of all dairy cattle during Second World War (Jasiorowski et al. 1988), for this study, a year 1950 was chosen as a base year for estimation of inbreeding level. At the beginning, the animals were mostly of a foreign origin, which was a consequence of absorptive crossing of Polish Black and White Lowland cattle (Jasiorowski et al. 1988; Goździkiewicz, 2004). This was observed especially in years 1950–1959, where over 90% of animals were imported yearly and those animals became the founders for the Polish Holstein-Friesian population. In 1970s, already several dozen Holstein-Friesian bulls were available for insemination, and from 1990s, bulls used for insemination have at least 87.5% of Holstein-Friesian blood (Goździkiewicz, 2004). Since 2010, the population in Poland has 93–99% of Polish Holstein-Friesians born yearly.

**Fig. 1:**
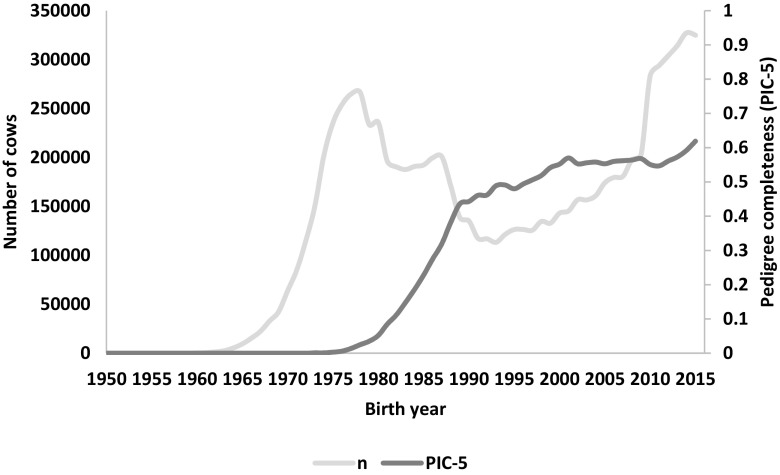
Number of cows and their pedigree completeness index based on five generations (PIC-5) obtained from pedigree of Polish Holstein-Friesian population

**Fig. 2:**
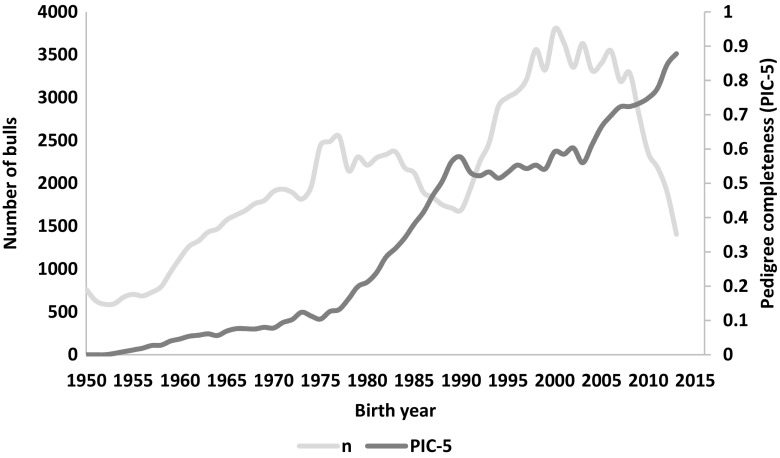
Number of bulls and their pedigree completeness index based on five generations (PIC-5) obtained from pedigree of Polish Holstein-Friesian population

**Fig. 3:**
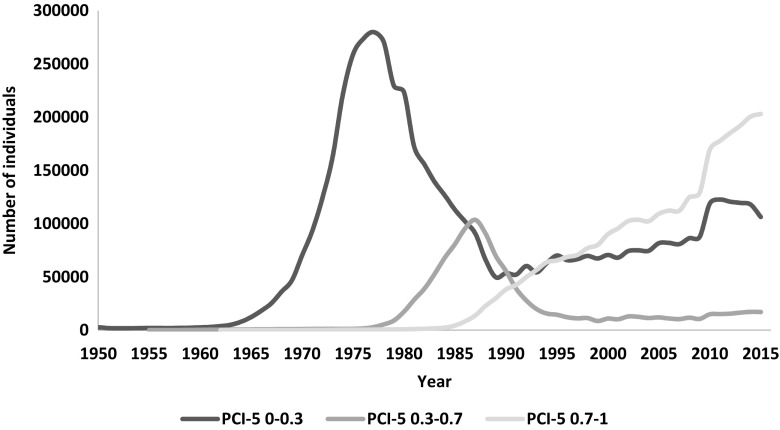
Number of individuals (cows and bulls) born per year in Polish Holstein-Friesian population presented for different levels of pedigree completeness index based on five-generations (PIC-5)

**Fig. 4:**
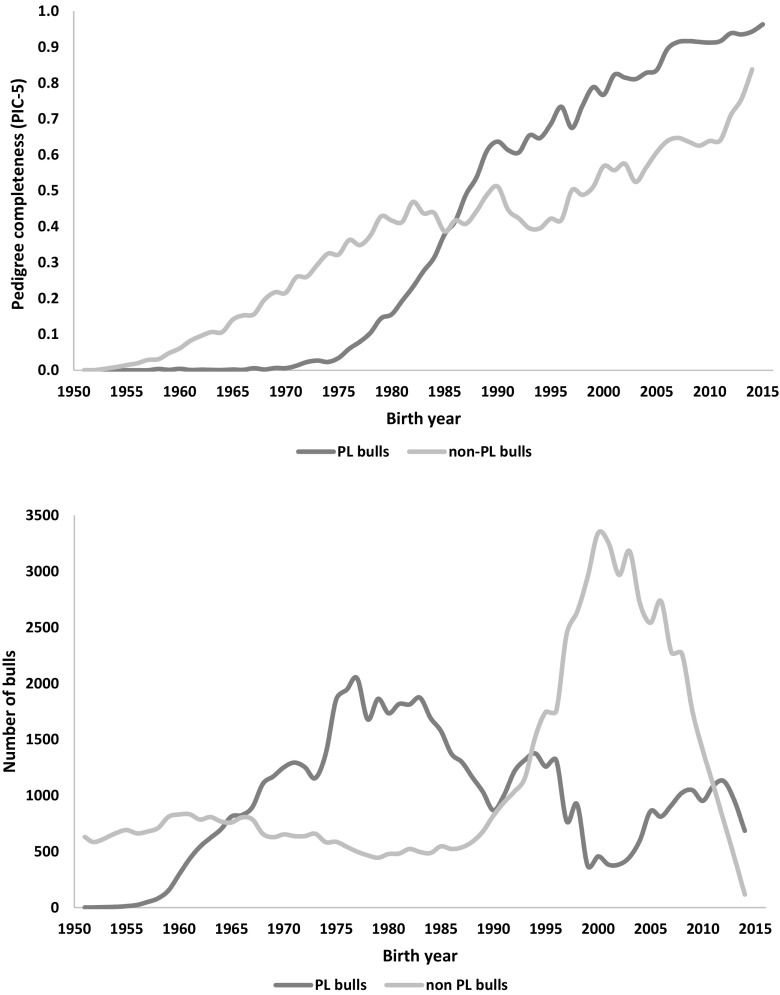
Pedigree completeness based on five generations of pedigree (PCI-5) and number of Polish (PL bulls) and foreign bulls (non-PL bulls) per birth year

**Fig. 5:**
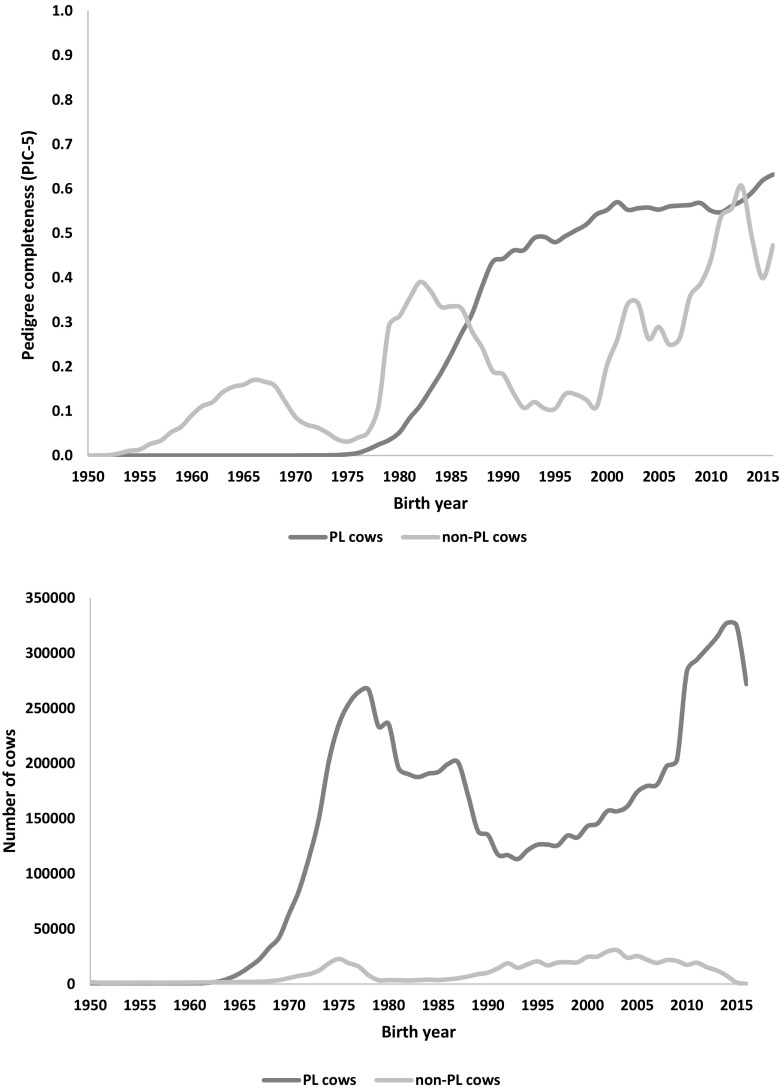
Pedigree completeness based on five generations of pedigree (PCI-5) and number of Polish (PL cows) and foreign cows (non-PL cows) per birth year

The pedigree completeness index (PIC-5) indicated that a large proportion of cows and bulls had none or scarce ancestral records until early 1980s (Figs. 1 and 2). However, from 1985, it was still observed that 30–50% of all animals (cows and bulls) being born each year had very low PIC-5 of 0.0–0.3 (Fig. 3). It has to be kept in mind that PCI-5 = 0 indicated that the animal had less than 1.5 of full generation recorded. Interestingly, the number of animals with a low PCI-5 continues to accure also in the most recent years 2010–2015 (Table 2). The Polish Holstein-Friesian population is still expanding in size, partially as a result of an import of foreign animals and semen (Figs. 4 and 5). Thus, it was assumed that a large number of animals without or with missing pedigree information were imported from other countries. However, further data evaluation indicated that the imported animals covered a very small proportion of those with PCI-5 = 0 (Table 2), even though their average PIC-5 was lower than that of Polish animals (Figs. 4 and 5). The cause for over 30% of all animals with a low pedigree completeness has its source in the fact that only ~36% of all the cows in Poland enter the milk recording evaluation. Even though each year the number of animals included in national milk recordings is increasing, a very large number of those cows is still without or with scarce pedigree information as they come from herds only lately included in the milk recordings.

**Table 2:** Comparison of the number of Polish and foreign Holstein-Friesian cattle with pedigree completeness index based on five generations equal to 0 (PCI-5 = 0)

Year of birth	All animals	Animals with PCI-5 = 0		
		All	Polish HF	Foreign HF
2010	300,980	112,651	105,060	7,591
2011	313,283	118,034	112,487	5,547
2012	319,327	115,541	111,428	4,113
2013	326,610	113,330	110,810	2,520
2014	334,826	110,817	108,027	2,790
2015	326,335	98,680	98,052	628

As for the year 2015 for cows and 2013 for bulls, the PCI-5 has been steadily increasing, and currently, it is on average 0.62 and 0.88, for cows and bulls, respectively (Figs. 1 and 2). Nonetheless, in comparison with other countries, it is a low result. For example, in Canada, Denmark, and UK, the PCI-5 on the level of 0.90 or higher is present in the local Holstein-Friesian populations already since early 2000s (Kearney et al. 2004; Sørensen et al. 2005; Stachowicz et al. 2011). The only solution to improve the pedigree completeness in Polish Holstein-Friesian population is raising awareness among farmers and breeders to keep accurate recordings on the matings performed in their herd. It has to be noted that underestimation of inbreeding level of an individual leads to overestimation of its breeding value and could result in selecting animals that are too related to the population. Only complete pedigree information will enable accurate estimation of relatedness within the population and with this, a more precise control of the change in inbreeding rate over time.

### Inbreeding levels

**Fig. 6:**
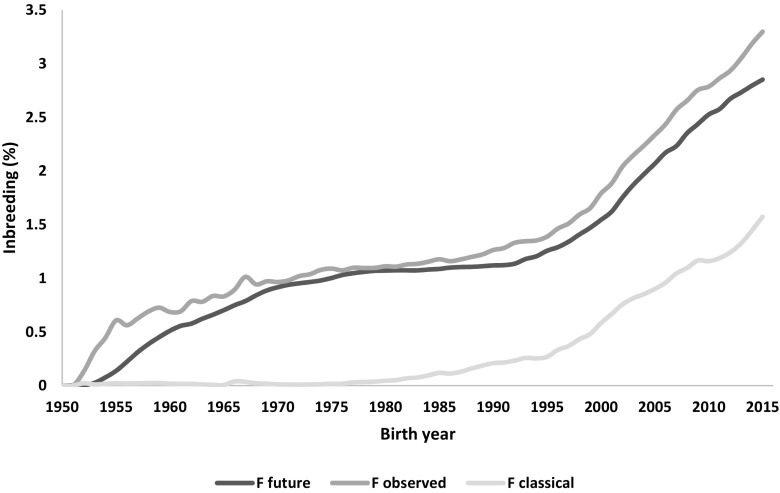
Comparison of inbreeding (F) estimated as F future, F observed, and F obtained with the classical approach (without accounting for missing parental information), based on pedigree for bulls and cows in Polish Holstein-Friesian population

Taking into account import of foreign cows (Fig. 4) and the relatively high import of foreign semen (Fig. 5) as well as very high number of new animals entering the milk recordings, evaluation of pedigree in such population is challenging. It is thus necessary to select a method computing the inbreeding coefficients with accounting for all information available in the pedigree and enabling estimation of the relationship level to the population of the animals with unknown parents. The method of VanRaden (1992) allows to assume that the animals without ancestors in the available pedigree have the inbreeding coefficient on the level of average inbreeding of the population from the birth year. The levels of inbreeding in the entire Polish Holstein-Friesian cattle population are presented in Fig. 6. The estimated level of the observed inbreeding for the end of 2015 was 3.30%. This value was achieved only because the applied method enables accounting for missing parent information (VanRaden 1992). Although the VanRaden’s algorithm (1992) was shown to recover most of information if 10–20% of dams were missing in the pedigree (Lutaaya et al. 1999), the classically estimated inbreeding for 2015 was only 1.56% (Fig. 6). This shows the robustness of the VanRaden’s algorithm and the ability to recover substantial amounts of missing pedigree data.

Nonetheless, to assure that the VanRaden’s algorithm (1992) applied to Polish pedigree does not overestimate the inbreeding of known foreign bulls, the values obtained in the present analysis were compared with the data from Canadian Dairy Network (CDN; https://www.cdn.ca). The comparison indicated that on average the estimates of inbreeding level for bulls (born in years 1952–2014) present on CDN website were by 1.46% higher than those from the evaluation of Polish Holstein-Friesian: 4.86 and 3.39%, respectively; whereas when the base year was not applied to the Polish dataset, the estimates were on average by 1.96% higher than the value reported in the international database, reaching 6.82%. The previous study estimating inbreeding in Polish Holstein-Friesian population suggested that using the base year is removing important part of information from the pedigree (Jankowski 2007). However, in this study, it can be concluded that the VanRaden’s algorithm (1992) accounts for most of the missing information in Polish pedigree. Also, using the base year is crucial to avoid overestimation of the inbreeding of foreign animals.

### Methodology vs. inbreeding estimation

Establishing the actual base year in any method remains debatable, as changing it directly affects the estimated level of inbreeding in the population, even though it does not affect the rate of increase in inbreeding over time. It was observed in the preliminary analysis that the change of rate inbreeding was not affected by the method selected nor the base year applied during the pedigree editing (results not shown). As presented in Fig. 6, also after establishing a base year and using three different methods for inbreeding estimation, its trend over time is very similar. This is also one of the WHFF’s assumptions and recommendations in the program monitoring the inbreeding in the world’s Holstein-Friesian populations, as the method and the level of inbreeding estimated with it is less important than the rate of increase in inbreeding over time (Fedderson et al. 2016). Thus, despite other countries using different methods to estimate the inbreeding levels in their Holstein-Friesian populations, e.g., Meuwissen and Luo (1992) in UK, Boichard (2002) in Dennmark, or Sargolzaei et al. (2005) in Canada, the rate of increase in inbreeding can be compared between them.

The inbreeding coefficient calculations in an ideal situation describe the actual inbreeding level in the population or of the single individual. However, when dealing with the actual pedigree data of the population, the calculations might be more or less further from the true values. Especially, in case of Polish pedigree, where the method accounting for missing parent information had to be used to recreate the relationship between the animals in the population, it is important to know that it did not affect the rate of increase in inbreeding. The only more precise method would be the estimation of the genomic inbreeding coefficient based on actual genotypic relationships between the animals (e.g., Leutenegger et al. 2003; VanRaden 2008; Bjelland et al. 2013). However, this is much more costly analysis than traditional pedigree-based inbreeding estimation and such data is not yet available for the Polish Holstein-Friesian population. Therefore, it needs to be assumed that despite its imperfections, assigning the average level of inbreeding from birth year to animals with unknown parents provides an inbreeding level closer to its actual value in the population and with appropriate trend over time.

### Observed vs. future inbreeding levels in polish population

**Fig. 7:**
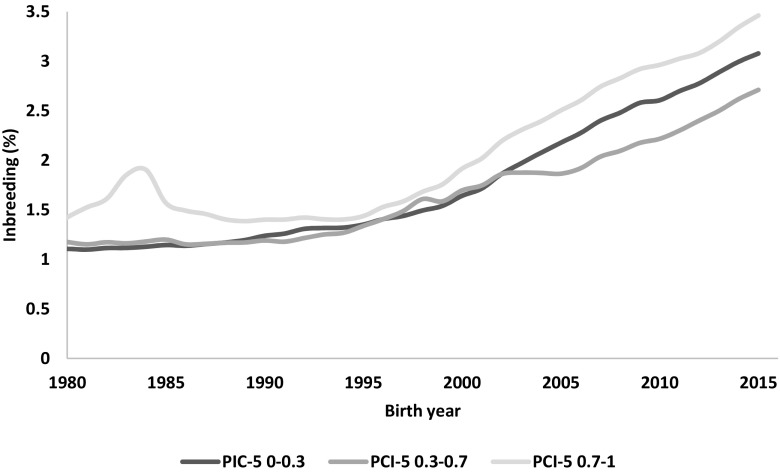
Observed inbreeding levels estimated based on pedigree for bulls and cows in Polish Holstein-Friesian population presented for different levels of pedigree completeness index based on five generations (PCI-5)

Interestingly, the future inbreeding was estimated on the level of 2.88% (Fig. 6). This means that the inbreeding estimated as an average of animals available for matings in a particular year is actually lower than its observed level. Moreover, the future inbreeding from year 1990 is constantly lower than observed inbreeding. Similar evaluation performed in USA by Council of Dairy Cattle Breeding (www.uscdbc.com) based on VanRaden and Smith (1999) showed different relation between future and observed inbreeding. In this population, the estimated future inbreeding was by 0.5–1% higher than estimated observed inbreeding until year 2015. Since then, it is lower by ~0.2% than level of observed inbreeding, which is most probably caused by intensification of genomic selection. It is possible that the differences between Polish and American Holstein-Frisian populations were caused by the Polish dairy cattle breeders, neglecting the importance of low inbreeding levels for production and reproduction traits and using bulls highly related to the population for their high breeding values despite possible negative impact of such matings. Furthermore, the Council of Dairy Cattle Breeding publishes frequently the list of bulls highly related to their population to be avoided in future matings. Poland does not provide such information yet to local farms. Also, the milk recordings of Holstein-Friesian cattle in Poland is still expanding, so it is clear that further selection is needed to improve production, which, if not monitored, can lead to increase in inbreeding levels. Thus, despite the estimates of inbreeding in Polish population being quite low, the difference between observed and future inbreeding indicates that it is very important to control the rate of increase in inbreeding over time to avoid further rapid increase. In addition, keeping in mind the number of animals with low value of PCI-5, if for inbreeding evaluation only animals with highly complete pedigrees were used the value of inbreeding would be 3.5% (Fig. 7). It could be hypothesized that the actual level of homozygosity in the Polish Holstein Friesian population is thus underestimated.

**Fig. 8:**
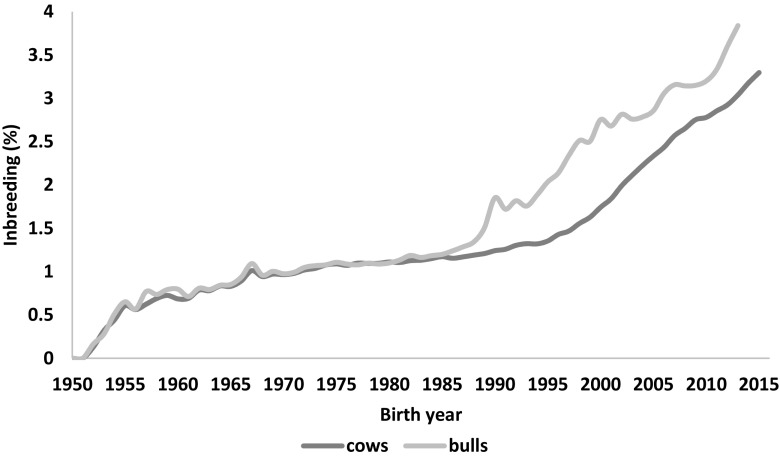
Inbreeding trend in Polish Holstein-Friesian population presented for bulls and cows separately

When comparing inbreeding levels in cows and bulls separately, the cows inbreeding for year 2015 is by 0.8% lower than for bulls (Fig. 8). This is also observed in other populations, e.g., USA (Council of Dairy Cattle Breeding; www.uscdbc.com). It might be caused by higher pedigree completeness and with this, more accurate estimates of inbreeding. Especially that in Poland, 42% of all inseminations are done with imported semen (report for the first half of 2015; Krychowski and Nowosielska 2016) and pedigree of such bulls is usually very deep. In addition, the inbreeding level obtained for cows was on the level expected from the complex study performed earlier (Jankowski 2007). Although this work covered data of cows born before 2005, similar values were obtained for the comparable periods of time: 2.23% in this study and 2.7% in Jankowski (2007). However, the inbreeding of bulls presented by him was much higher (4.2% in 2004; Jankowski 2007) than observed in this study (2.6% in 2004). The difference in two studies was that Jankowski (2007) did not use the base year and since for the bulls the pedigrees were on general deeper this could have included excessive information leading to overestimation of the inbreeding level.

### Comparison of polish population with other countries

**Fig. 9:**
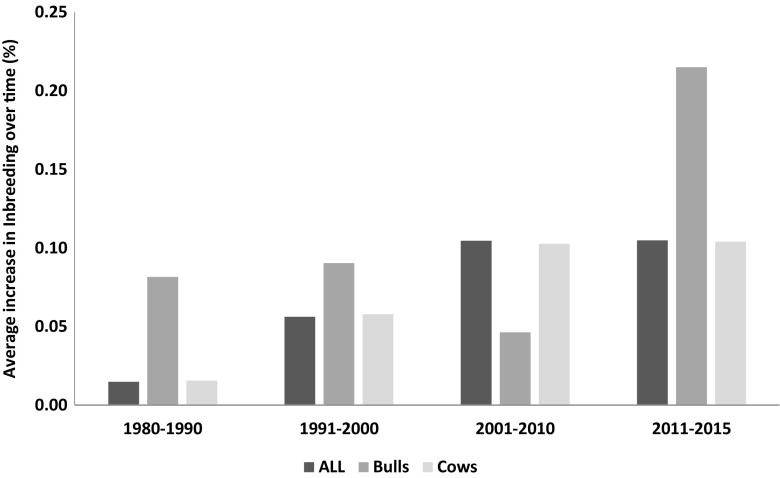
Average yearly rate of increase in inbreeding level in Polish Holstein-Friesian population in 10-year time frames for all animals, bulls, and cows

The inbreeding level estimated for 2015 would place Polish population in the penultimate position in the international comparison of WHFF, between Holstein-Friesian populations from Israel (~2.5% in year 2013; Fedderson et al. 2016) and Belgium (~3.5% in year 2015; Fedderson et al. 2016). Also, the average rate of increase in inbreeding over time in Polish population is low—0.10% (Fig. 9). Populations in five countries, such as the Netherlands (0.07%) or Japan (0.09%), have reported lower increase of inbreeding, but in nine countries, the average increase in inbreeding was higher, e.g., USA (0.17%) or Canada (0.21%). Despite a more rapid increase in 2000s, the values obtained for Polish population indicate very low increase of inbreeding over the past decades, which is expected to be below 1% per generation (Falconer and Mackay 1996; Lynch and Walsh 1998).

The reason for these low values could be firstly the fact that Polish population is still importing nearly half of used semen and high number of animals from other countries. This means that foreign genetic material is constantly added to the Polish Holstein-Friesian population. Naturally, those animals or semen are not outcrossed in comparison to Polish cattle, as the globalization of Holstein-Friesian cattle is progressing, but have other pedigree lines than local population, which affects the estimation of inbreeding level. Secondly, quite low estimates for Polish population are directly linked with pedigree structure, depth, and low completeness that are most probably causing underestimation of the inbreeding level. Even use of the method allowing for accounting for missing parental information cannot recover it fully. Nonetheless, the rate of increase in inbreeding over time remains the same despite the applied method (VanRaden vs. classical approach).

Another step that has to be taken into consideration in inbreeding level control of Polish Holstein-Friesian population is the estimation of genomic inbreeding (Wiggans et al. 1995; VanRaden et al. 2011). Such analyses are performed in USA on regular bases since 2009 by Council of Dairy Cattle Breeding (www.uscdbc.com) and indicate far higher levels of genomic (~10.2%; www.uscdcb.com 2016) than pedigree-based inbreeding (~7.7%; www.uscdcb.com 2016). To implement genomic evaluation of inbreeding level in Polish population, the sufficient number of genotypes in necessary. As the number of genotyped Holstein-Friesian cattle in Poland is low, it is crucial to collaborate with organizations such as EuroGenomics to allow implementing the new methodology together with other countries, which should be the next step in inbreeding control.

## Conclusions

This study aimed to estimate observed and future inbreeding levels in Polish Holstein-Friesian population. This was required to enable the control of rate of increase in inbreeding over time. The obtained values indicated that the inbreeding in Polish population is on a quite low level and that its increase over the past decades remains within the value recommended by WHFF. However, the values might be underestimated due to low level of pedigree completeness. The estimates of future inbreeding suggested that the level of observed inbreeding could be even lower and also increase over time slower, which indicates the need to monitor rate of increase in inbreeding over time. The most important aspect of the presented results is the necessity to advise individual farmers to keep precise recordings of the matings on their farm in order to improve the pedigree completeness of Polish Holstein-Friesian and to use suitable mating programs to avoid too rapid growth of inbreeding. For the future, an application of genomic data to estimate genome-based inbreeding coefficient seems to be the best option to avoid issues with pedigree quality.

## References

[CR1] Aguilar I, Misztal I (2008). Technical note: recursive algorithm for inbreeding coefficients assuming nonzero inbreeding of unknown parents. J Dairy Sci.

[CR2] Bjelland DW, Weigel KA, Vukasinovic N, Nkrumah JD (2013). Evaluation of inbreeding depression in Holstein cattle using whole-genome SNP markers and alternative measures of genomic inbreeding. J Dairy Sci.

[CR3] Boichard, D (2002) Pedig: a Fortran package for pedigree analysis suited for large populations proc. 7th world Congr Genet Appl Livest Prod, Montpellier, France

[CR4] Emik LO, Terrill CE (1949). Systematic procedures for calculating inbreeding coefficients. J Hered.

[CR5] Falconer DS, Mackay TFC (1996). Introduction to quantitative genetics.

[CR6] Feddersen E, Van Doormal B, de Jong G, Rensing S (2016). World inbreeding trend in Holsteins.

[CR7] Jankowski, T. (2007) Optimization of mating design with constrained inbreeding of Polish Holstein-Friesian. PhD Thesis. Poznan university of life sciences

[CR8] Jasiorowski, H. A., M. Stolzman, and Z. Reklewski (1988) The international Friesian strain comparison trial. A world perspective. Food and agriculture organization of the United Nations

[CR9] Kearney JF, Wall E, Villanueva B, Coffey MP (2004). Inbreeding trends and application of optimized selection in the UK Holstein population. J Dairy Sci.

[CR10] Krychowski, T., and A. Nowosielska. 2016. Analiza użytkowania nasienia buhajów w pierwszym półroczu 2015 roku. 2:4–8

[CR11] Goździkiewicz L (2004). 100 lat oceny wartości użytkowej bydła w Polsce.

[CR12] Lal C, Kumar V, Maloo SR (2013). Heterosis and inbreeding depression for some quantitative and heat tolerance characters in bread wheat (*Triticum aestivum* L.). J Wheat Res.

[CR13] Leutenegger AL, Prum B, Génin E, Verny C, Lemainque A, Clerget-Darpoux F, Thompson EA (2003). Estimation of the inbreeding coefficient through use of genomic data. Am J Hum Genet.

[CR14] Lutaaya BE, Misztal I, Bertrand JK, Mabry JW (1999). Inbreeding in populations with incomplete pedigrees. J Anim Breed Genet.

[CR15] Lynch M, Walsh B (1998). Genetics and analysis of quantitative traits.

[CR16] MacCluer JW, Boyce AJ, Dyke B, Weitkamp LR, Pfenning DW, Parsons CJ (1983). Inbreeding and pedigree structure in Standardbred horses. J Hered.

[CR17] Meuwissen THE, Luo Z (1992). Computing inbreeding coefficients in large populations. Genet Sel Evol.

[CR18] Miglior F, Burnside EB (1995). Inbreeding of Canadian Holstein cattle. J Dairy Sci.

[CR19] Rokouei M, Torshizi RV, Shahrbabak MM, Sargolzaei M, Sørensen AC (2010). Monitoring inbreeding trends and inbreeding depression for economically important traits of Holstein cattle in Iran. J Dairy Sci.

[CR20] Sargolzaei M, Iwaisaki H, Colleau JJ (2005). A fast algorithm for computing inbreeding coefficients in large populations. J Anim Breed Genet.

[CR21] Sørensen AC, Sørensen MK, Berg P (2005). Inbreeding in Danish dairy cattle breeds. J Dairy Sci.

[CR22] Strabel T (2001). Ograniczanie inbredu we współczesnej hodowli bydła mlecznego. Prace i Materiały Zootechniczne.

[CR23] Stachowicz K, Sargolzaei M, Miglior F, Schenkel FS (2011). Rates of inbreeding and genetic diversity in Canadian Holstein and Jersey cattle. J Dairy Sci.

[CR24] VanRaden PM, Olson K, Wiggans G, Cole JB, Tooker ME (2011). Genomic inbreeding and relationships among Holsteins, jerseys, and Brown Swiss. J Dairy Sci.

[CR25] VanRaden PM (1992). Accounting for inbreeding and crossbreeding in genetic evaluation of large populations. J Dairy Sci.

[CR26] VanRaden PM, Smith LA (1999). Selection and mating considering expected inbreeding of future progeny. J Dairy Sci.

[CR27] VanRaden PM (2005). Inbreeding adjustments and effect on genetic trend estimates. Interbull Bull.

[CR28] VanRaden PM (2008). Efficient methods to compute genomic predictions. J Dairy Sci.

[CR29] Wiggans GR, VanRaden PM, Zuurbier J (1995). Calculation and use of inbreeding coefficients for genetic evaluation of United States dairy cattle. J Dairy Sci.

[CR30] Wright S (1922). Coefficients of inbreeding and relationship. Am Nat.

[CR31] Young CW, Seykora AJ (1996). Estimates of inbreeding and relationship among registered Holstein females in the United States. J Dairy Sci.

